# Amorphous Polymer–Phospholipid Solid Dispersions for the Co-Delivery of Curcumin and Piperine Prepared via Hot-Melt Extrusion

**DOI:** 10.3390/pharmaceutics16080999

**Published:** 2024-07-28

**Authors:** Kamil Wdowiak, Andrzej Miklaszewski, Judyta Cielecka-Piontek

**Affiliations:** 1 Department of Pharmacognosy and Biomaterials, Poznan University of Medical Sciences, 3 Rokietnicka St., 60-806 Poznan, Poland; kamil.wdowiak@student.ump.edu.pl; 2 Institute of Materials Science and Engineering, Poznan University of Technology, Jana Pawla II 24, 61-138 Poznan, Poland; andrzej.miklaszewski@put.poznan.pl

**Keywords:** curcumin, piperine, amorphous solid dispersion, phospholipids, hot-melt extrusion, supersaturation, solubility-enabling formulation, bioavailability

## Abstract

Curcumin and piperine are plant compounds known for their health-promoting properties, but their use in the prevention or treatment of various diseases is limited by their poor solubility. To overcome this drawback, the curcumin–piperine amorphous polymer–phospholipid dispersions were prepared by hot melt extrusion technology. X-ray powder diffraction indicated the formation of amorphous systems. Differential scanning calorimetry confirmed amorphization and provided information on the good miscibility of the active compound–polymer–phospholipid dispersions. Owing to Fourier-transform infrared spectroscopy, the intermolecular interactions in systems were investigated. In the biopharmaceutical properties assessment, the improvement in solubility as well as the maintenance of the supersaturation state were confirmed. Moreover, PAMPA models simulating the gastrointestinal tract and blood-brain barrier showed enhanced permeability of active compounds presented in dispersions compared to the crystalline form of individual compounds. The presented paper suggests that polymer–phospholipid dispersions advantageously impact the bioaccessibility of poorly soluble active compounds.

## 1. Introduction

The poor solubility of active pharmaceutical agents is a major obstacle to the search for novel oral formulations and causes many promising new drugs to be rejected at an early stage of development due to poor performance in in vivo studies [1]. Currently, many researchers are focusing their efforts on creating delivery systems or formulations to improve solubility. Among the numerous solutions to the problem of poor bioaccessibility are self-emulsifying drug delivery systems, the formation of inclusion complexes with cyclodextrin, obtaining nanoparticles, and the formation of co-crystals [2,3,4].

The problem of developing formulations of active substances with increased solubility is also evident in the manufacturing of nutraceuticals, defined as a food or food component with health benefits [5]. Curcumin and piperine are the main active compounds of the popular spices turmeric (*Curcuma longa* L.) and black pepper (*Piper nigrum* L.). These compounds exhibit beneficial health-promoting properties that can be used in the prevention and treatment of many diseases in civilization. Therefore, they can be considered as nutraceuticals. Poor solubility is a limiting factor in the activity of many plant-derived compounds, such as curcumin and piperine. Curcumin has been reported to show several health-promoting properties, such as antioxidant, anti-inflammatory, anti-cancer, anti-diabetic, neuroprotective, and cardioprotective activity [6,7,8]. Piperine, an alkaloid compound, has been reported to exhibit anti-oxidant, anti-inflammatory, anti-carcinogenic, anti-diabetic, anti-obesity, and neuroprotective activity [9,10,11].

One promising formulation approach is the use of an amorphous form of the active ingredient. The amorphous form is characterized by a lack of long-range ordering and, therefore, a lack of crystal structure. This makes the dissolution of the substance in the amorphous form not require the energy inputs needed to release the substance from the crystal structure, so better solubility of the amorphous substance is observed [12]. The use of the amorphous form is associated with the need to ensure product stability. The amorphous form has a natural tendency to transform into a more stable crystalline form, which will result in the loss of the favorable effect on solubility [1]. Thus, it is crucial to reduce the risk of crystallization. The addition of excipients is aimed at providing amorphous form stability. One possible solution is to use a polymer as a matrix in which the active substance is dispersed [13].

Another well-established approach to improving bioavailability is the use of phospholipids. Phospholipids are biocompatible structures composed of a hydrophilic head and a hydrophobic chain, which makes them exhibit an amphiphilic nature. Thanks to their properties of self-organization, emulsification, and wetting, they are used not only in obtaining drug carriers but also as emulsifiers or surfactants [14,15]. Phospholipids can be used in parenteral, oral, or topical formulations [16].

Considering the potential benefits of combining the stabilization of an amorphous form using polymers and phospholipids, this work aimed to develop amorphous dispersions of a polymer–phospholipid and curcumin and piperine to improve the bioaccessibility of these plant-derived compounds. The hot-melt extrusion technology was employed as a method to obtain amorphous dispersions. At first, we established the plasticizing effect of the phospholipid on polyvinylpyrrolidone (PVP) K25 and designed the composition of the polymer–phospholipid blend. In the second stage of the research, we focused on producing and characterizing curcumin–piperine systems dispersed in the polymer–phospholipid matrix.

## 2. Materials and Methods

### 2.1. Materials

Piperine (purity > 95%, FG) was acquired from Sigma-Aldrich (Sigma-Aldrich, St. Louis, MO, USA), whereas curcumin (purity > 95%) was bought from Xi’an Tian Guangyuan Biotech Co., Ltd. (Xi’an, China). Excipients were provided by the following manufacturers: PVP K25 by JRS Pharma (Rosenberg, Germany), xylitol by Santini (Poznań, Poland), and phosphatidylcholine (from soybean, Type II-S, 14–29% choline basis, purity 21%) by Sigma-Aldrich (St. Louis, MO, USA). The other reagents used were sodium hydroxide (Avantor Performance Materials Poland S.A., Gliwice, Poland), acetic acid (98–100%; POCH, Gliwice, Poland), sodium dimethyl sulfoxide (DMSO; PanReac Appli-Chem ITW Reagents, Darmstadt, Germany), acetic acid (J. T. Baker, Center Valley, PA, USA), and HPLC-grade methanol (J. T. Baker, Center Valley, PA, USA). Using a Direct-Q 3 UV purification system (Millipore, Molsheim, France; model Exil SA 67120), high-grade, laboratory-grade clean water was produced. The suppliers of the Prisma HT, GIT/BBB lipid solution, and acceptor sink buffer were Forest Row, East Sussex, UK-based Pion Inc.

### 2.2. Methods

#### 2.2.1. Preparation of Polymer–Phospholipid/Polymer–Xylitol Mixtures

Polymer–phospholipid/polymer–xylitol blends were prepared by dissolving appropriate amounts of ingredients needed to obtain 10 g of the blend in 100 mL of purified water (T = 22 °C). To determine the plasticizing effect, blends with different concentrations of (*w*/*w*) phospholipids and xylitol were prepared. Based on the determined equations of the linear function, the amount of excipient needed to obtain a polymer–phospholipid–xylitol blend with a glass transition value of 120 °C was calculated. To prepare the polymer–phospholipid–xylitol mixture with a glass transition temperature of 120 °C, the excipients were weighed, then transferred to a beaker and dissolved in 100 mL of purified water and stirred on a magnetic stirrer; then, the colloidal dispersion was lyophilized (LyoQuest-85, Telstar, Terrassa, Spain) to obtain a powder. The freeze-drying process was carried out at a temperature of −82.1 °C and under vacuum at a pressure of 0.412 mbar. The freeze-dried products were powdered using a mill (Tube Mill Control Mixer, IKA, Warsaw, Poland).

#### 2.2.2. Preparation of Hot-Melt Extruded Systems

**Figure 1:**
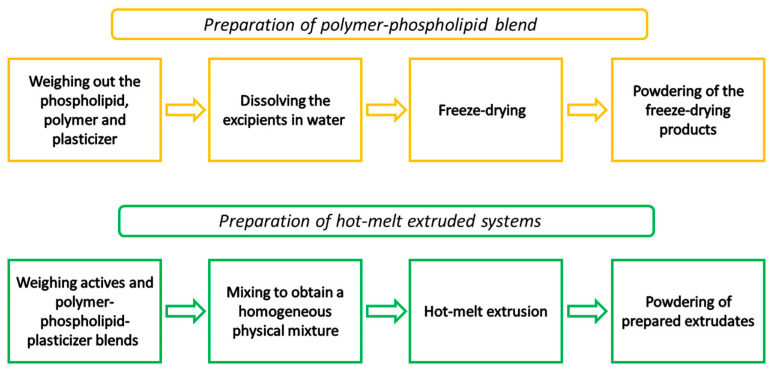
Scheme illustrating the preparation of polymer–phospholipid–plasticizer blends and hot-melt extruded systems.

A HAAKE MiniCTW micro-conical twin screw extruder (Thermo Scientific, Karlsruhe, Germany) was used to perform the hot-melt extrusion procedure. The polymer–phospholipid–xylitol or polymer–xylitol blends were mixed with 15 and 30% total content of active compounds (1:1 curcumin: piperine mass ratio) using a mill (Tube Mill Control Mixer, IKA, Warsaw, Poland). The physical mixes were then manually placed into the extruder’s hopper while the screw was spinning at 90 rpm and the barrel temperature was 150 °C. The prepared extrudates were powdered by milling (Tube Mill control Mixer, IKA, Warsaw, Poland), sifted through a 0.355 mm sieve, and used for further studies. The preparation procedure of the polymer–phospholipid–plasticizer blends is presented in Figure 1. The composition of the formulations is shown in Table 1. 

#### 2.2.3. X-ray Powder Diffraction (XRPD)

The samples’ diffraction patterns were acquired using X-ray diffraction (XRD, Pan-analytical Empyrean, Almelo, The Netherlands) equipment with the copper anode (Cu-Kα—1.54 Å, 45 kV and 40 mA) in the Bragg–Brentano reflection mode configuration. The measurement parameters were adjusted to 5–60° 2 theta, with a 45 s step per 0.05°.

#### 2.2.4. Differential Scanning Calorimetry (DSC)

A DSC 214 Polyma differential scanning calorimeter (Netzsch, Selb, Germany) was used for the thermal analysis. Samples of about 5–10 mg were put into crimped aluminum pans with a small hole in the lid. To remove water from the samples, they were heated to 80 °C and kept for 8 min at this temperature, cooled to 25 °C, and then heated again to 235 °C. Raw compounds were heated to 235 °C, cooled to 25 °C, and then heated once again to 235 °C to determine their glass transition temperature (Tg). The experiments were conducted in a nitrogen atmosphere with a flow rate of 30 mL/min, with heating and cooling rates of 10 °C/min and 10 °C/min, respectively. The Tg was taken as the midpoint between onset and endpoint temperatures.

Applying a heating–cooling–heating cycle, the Tgs values of PVP K25–phospholipid/PVP K25–xylitol mixtures were examined. To remove any remaining water, the first heating was performed. The sample was heated to 200 °C at a rate of 20 °C/min, cooled to 25 °C at a rate of 10 °C/min, and heated again to 200 °C at a rate of 20 °C/min.

Various heating–cooling–heating cycles were carried out to evaluate the glass-forming abilities. The sample, weighing 5–10 mg, was first heated to a temperature of about 10 °C over the compound’s melting point. It was then cooled to 25 °C at various rates (20, 10, and 5 °C/min), and heated again at a rate of 20 °C/min to a temperature of 10 °C above the melting point.

#### 2.2.5. Fourier-Transform Infrared Spectroscopy (FTIR-ATR)

A Shimadzu IRTracer-100 spectrometer equipped with a QATR-10 single bounce, a diamond extended range, and LabSolutions IR software (version 1.86 SP2, Warsaw, Poland) was used to measure FTIR-ATR spectra at a resolution of 1 cm−1. Compounds were produced in amorphous forms using DSC, as previously mentioned in the section on differential scanning calorimetry.

#### 2.2.6. Physical Stability

The physical stability was observed for up to 9 months. Storage conditions were as follows: (1) 40 °C and 75% relative humidity; (2) 30 °C and 65% relative humidity; (3) 50 °C and dry conditions provided by silica gel; (4) 25 °C and dry conditions. Conditions with specific humidity (1, 2) were provided by climatic chambers Memmert OC H260L (Memmert GmbH, Büchenbach, Germany). The samples were taken out after 9 months and examined for the presence of crystallinity by XRPD. The samples were stored in a light-protected condition in glass vials.

#### 2.2.7. High-Performance Liquid Chromatography (HPLC) Analysis

HPLC was used to measure the concentrations of piperine and curcumin in samples taken during permeability, dissolution, and solubility investigations. A Shimadzu LC-2050C (Shimadzu Corp., Kyoto, Japan) was equipped with a diode array detector (DAD). A Dr. Maisch ReproSil-Pur Basic-C18 100 Å column with a 5 µm particle size and a 100 × 4.60 mm size (Dr. Maisch, Ammerbuch-Entringen, Germany) was used as a stationary phase. HPLC-grade methanol/0.1% acetic acid (85:15 *v*/*v*) was used as the mobile phase. The mobile phase was vacuum filtered through a 0.45 µm nylon filter (Phenomenex, Torrance, CA, USA). The analysis parameters were as follows: a flow rate of 0.3 mL/min, a wavelength of 420 nm for curcumin and 340 nm for piperine, and a column temperature of 30 °C. The injection volume differed depending on the assay. For the solubility study, it was 2 µL, and for the dissolution and permeability assays, it was 10 µL. The duration of the run was 9 min. The retention times for curcumin and piperine were 4.912 min and 5.851 min, respectively. Chromatograms (Figure S1) and method validation parameters (Table S1) were added to the Supplementary Materials.

#### 2.2.8. Preparation of Media for Solubility Studies and Dissolution

The following steps were used to prepare phosphate buffer at pH 6.8: In a 1000 mL volumetric flask, 250 mL of 0.2 N potassium dihydrogen phosphate solution was added, followed by 112 mL of a 0.2 N sodium hydroxide solution. Purified water was added to make the volume up to 1000 mL. A hydrochloric acid solution with a pH of 1.2 was prepared according to the manufacturer’s instructions (Alfachem, Poznań, Poland) from the analytically weighed amount.

#### 2.2.9. Solubility Studies

A 10 mL glass tube was filled with an excess amount of powder (corresponding to 4 mg of each active substance). Then, 2.0 mL of phosphate buffer (pH 6.8) or HCl solution (pH 1.2) was added and stirred at 100 rpm at room temperature (25 ± 0.1 °C) for 24 h. The obtained solutions were filtered, diluted 1:20 *v*/*v* with water, and filtered again through a 0.2 μm PTFE membrane filter (Sigma-Aldrich, St. Louis, MO, USA) and analyzed using the HPLC method described above.

#### 2.2.10. Dissolution Studies

The paddle apparatus (Agilent 708-DS dissolution apparatus, Santa Clara, CA, USA) was used to conduct the dissolution study. A gelatin capsule (size: 1) was filled with the compound and extruded systems in an amount equal to 5 mg of each active ingredient. The capsule was then put in a spring that served as a sinker and added to the dissolution medium. The vessels were filled with 500 mL of phosphate buffer at a pH of 6.8 or HCl at a pH of 1.2; the temperature was kept at 37 ± 0.1 °C, and the paddle speed was set to 50 rpm at the following time intervals: 5 min, 15 min, 30 min, 1 h, 2 h, 3 h, 4 h, 5 h, and 6 h. Aliquots of 2.0 mL of liquid were taken out and replaced with equal volumes of temperature-equilibrated media. Samples were then filtered through a 0.2 μm PTFE membrane filter, and HPLC analysis was performed. Each sample was tested 3 times.

#### 2.2.11. Permeability Studies

The Parallel Artificial Membrane Permeability Assay (PAMPA) models were used to study the permeability of the blood-brain barrier (BBB) and the gastrointestinal tract (GIT) in vitro. Two 96-well microfilter plates formed the sandwich. There were two chambers in the PAMPA system: the acceptor chamber at the top and the donor chamber at the bottom. The chambers were separated by a 120 μm thick PVDF membrane coated with a 20% (*w*/*v*) dodecane solution of a lecithin mixture (Pion, Inc., Forest Row, East Sussex, UK). The manufacturer provided the acceptor and donor solutions. As specified by the manufacturer, the donor solution was diluted, and then 0.5 M of NaOH was used to adjust the pH to ≈6.8 for the GIT assay and ≈7.4 for the BBB assay. After combining the plates, both models were incubated for four hours at 37 °C in a humidity-saturated environment. The samples for the donor compartments were first prepared in the same manner as the solubility studies using purified water. Then, the systems were diluted 1:5 *v*/*v* with water, filtered through a 0.2 μm PTFE membrane filter, and further diluted 1:1 *v*/*v* with DMSO. Next, the obtained solutions were placed into the donor compartments after being diluted 1:1 *v*/*v* with the donor solution for the GIT and BBB tests. The compound’s concentration in the acceptor solution was used to express the results.

#### 2.2.12. Statistical Analysis

Results are expressed as the mean ± standard deviation. Statistical tests were performed using a one-way analysis of variance (ANOVA), and statistical differences were determined using Duncan’s tests with a significance threshold of *p* < 0.05. All statistical analyses were performed using Statistica 13.1 software (TIBCO Software Inc., Palo Alto, CA, USA).

## 3. Results and Discussion

In this work, we attempted to fabricate and characterize amorphous polymer–phospholipid dispersions. Amorphous polymer–phospholipid dispersions can combine the advantages of phospholipids with the beneficial effects of amorphization on solubility. This formulation approach will be particularly desirable for poorly soluble substances, as solubility limits their therapeutic potential. Moreover, it has been indicated that amorphous dispersions, by providing increased solubility, also boost drug permeability by raising the concentration of free, absorbable molecules, which drives transport from the intestinal lumen to enterocytes [17]. Beig et al.’s research demonstrated the superiority of amorphous dispersion over other techniques for improving bioaccessibility. The authors indicated that the amorphous dispersion of etoposide increased the solubility of the active ingredient while increasing permeation. In contrast, approaches based on the use of cyclodextrin, a solubilizer, or a cosolvent increased solubility but decreased permeation of etoposide [18]. This study was conducted in a PAMPA model simulating passive diffusion, one of the essential drug absorption routes [19].

There is evidence that the addition of phospholipids to the formulation increases permeability and oral bioavailability. Ma et al. obtained a mangiferin–phospholipid complex, which showed enhanced intestinal permeability, leading to a higher plasma concentration of mangiferin in rats after oral administration [20]. Moreover, the addition of phospholipids appears to be an effective way to boost the bioavailability of curcumin. Maiti et al. showed that the curcumin–phospholipid combination improved the curcumin’s solubility and the bioavailability of curcumin in rats, which translated into an improved hepatoprotective activity of curcumin [21].

Phospholipids can cause technological challenges as there is a risk of obtaining a system with a lipidic texture that is unsuitable for solid dosage forms. Hence, they are often combined with an additional excipient as a stabilizer to obtain a powder. Jo et al. used Neusilin^®^ US2 to powderize a phosphatidylcholine–celecoxib dispersion [22]. On the other hand, Brinkmann-Trettenes et al. used trehalose to obtain a solid powder of phospholipid by employing a spray-drying technique [23]. Moreover, phospholipid complexes with active agents provide enhanced solubility and promote absorption. However, they tend to aggregate and agglomerate, which adversely affects the dissolution and absorption of therapeutic substances. Hence, it is important to find strategies to eliminate these adverse phenomena so that phospholipids can be used in oral delivery on a larger scale [24]. An approach combining an amorphous polymer–phospholipid dispersion was used by Zhou et al. They used PVP K30 to disperse the baicalein–phospholipid complex. The authors demonstrated that the polymer–phospholipid combination matrix showed higher levels of dissolved baicalein than the baicalein–phospholipid complex itself. Moreover, the polymer–phospholipid matrix ensured better bioavailability of baicalein after oral administration to rats, which the authors attributed to several factors, including obtaining an amorphous form of baicalein, the permeability enhancing property of phospholipid, not destroying the complexation state of baicalein and phospholipid, and providing high dispersibility of the baicalein–phospholipid complex by adding the PVP K30 matrix [24]. This paper supports the development of polymer–phospholipid formulations, presenting them as superior to simple active substance–phospholipid complexes. In this work, we suggested combining a phospholipid with a polymer, an approach to introducing phospholipids as an additive in oral formulations and presenting them in a form that will allow the employment of hot-melt extrusion technology.

Curcumin and piperine were obtained as a co-amorphous system by Wang et al. [25]. The authors demonstrated the formation of hydrogen bonds in the systems, as well as an increase in the permeability in the Caco-2 model and apparent solubility. However, the systems were obtained via a quench-cooling approach, which cannot be used on a larger scale, unlike hot-melt extrusion. Furthermore, the literature indicates that amorphous dispersions using polymers or solubilizers show better biopharmaceutical properties than drug–drug co-amorphous combinations alone. Amorphous polymeric dispersions achieve higher apparent solubility values than substances in amorphous form without an excipient, highlighting the importance of the polymer as a solubility enhancer that inhibits crystallization [26]. In addition, it is indicated that amorphous polymer dispersions provide better physical stability and bioavailability than co-amorphous systems [27]. These arguments justify obtaining amorphous dispersions with excipients as crystallization inhibitors. 

In our study, a fixed-dose formulation concept was developed to co-deliver curcumin and piperine. As defined, fixed-dose products are combinations of at least two active ingredients in a single dosage form [28,29,30]. Our approach was based on reports indicating that such a combination is superior to single compounds administered separately in terms of their bioavailability and therapeutic effect. From a bioavailability perspective, piperine is a well-documented bioenhancer. The co-administration of piperine leads to improved blood concentrations owing to efflux transporter inhibition as well as suppression of liver enzymes, reducing the first-pass effect [31,32]. From the therapeutic point of view, there are reports suggesting synergy between curcumin and piperine, boosting pharmacological effect, especially neuroprotective synergy [33,34,35].

It is worth mentioning that polymers of the PVP group have several grades due to different polymer chain lengths. It is known that as PVP grade increases, an increase in the ability to inhibit crystallization is observed [36], but an increase in Tg values is also observed [37]. Lower-grade polymers such as PVP K12 were rejected in this study due to concerns about the insufficient stabilizing ability of the amorphous form, affecting poorer biopharmaceutical performance. Higher-grade polymers exhibit higher Tg, and this would require higher amounts of plasticizer. PVP K25 was chosen as a compromise between its ability to inhibit crystallization and a Tg value that would not require a large amount of plasticizer.

**Figure 2:**
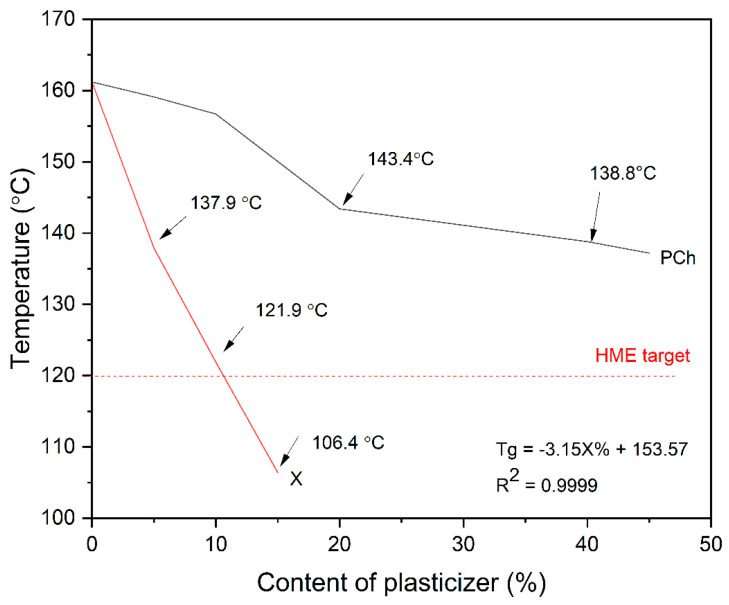
The dependence of the xylitol and phospholipid content on the Tg value of the PVP K25–excipient blend. The red dashed line shows the target Tg. Arrows point Tg. The X stands for xylitol, while PCh stands for phospholipid.

The hot-melt extrusion process was planned to be performed at 150 °C based on the experience of other research groups that used hot-melt extrusion to obtain curcumin systems [38]. Bearing in mind the hot-melt extrusion rule indicating that the process of extrusion needs to be run at a temperature at least 20 °C higher than the Tg of the polymer [39], we designed the carrier to have glass transition (Tg) of about 120 °C as a compromise between smooth processing and physical stability of obtained dispersions. In the first attempts to develop a polymer–phospholipid blend with the desired extrudability, an acceptable Tg value to allow extrusion under the planned temperature conditions, the plasticizing effect of a phospholipid was tested. Here, it is worth highlighting that we used phosphatidylcholine as a phospholipid representative. Differential Scanning Calorimetry (DSC) studies showed that phospholipid alone had an insufficient plasticizing effect on PVP K25 (Figure 1). The addition of 40% phospholipids to the polymer resulted in a decrease in Tg to a value of 138.8 °C, which forced us to introduce another excipient acting as a plasticizer into the carrier to achieve the desired Tg value. Based on previous experience, we decided to choose xylitol as a plasticizer [40]. Xylitol showed a significant plasticizing effect on PVP K25 (Figure 2). Knowing the effect of a phospholipid on the Tg of PVP K25 and based on the plot of the linear function, we could determine the final composition of the polymer–phospholipid–plasticizer blend with the desired extrudability.

After we optimized our carrier for the desired thermal properties, we were able to continue with the manufacturing of active compound systems. In this paper, we introduce the following labels for the systems: X = system without phospholipid, PCh20 = system with a 20% addition of phospholipids in the carrier, PCh40 = system with a 40% addition of phospholipids in the carrier, and the number denotes the total content of curcumin and piperine in the system (mass ratio 1:1 *w*/*w*). In the next step, we focused on solid-state characterization. 

**Figure 3:**
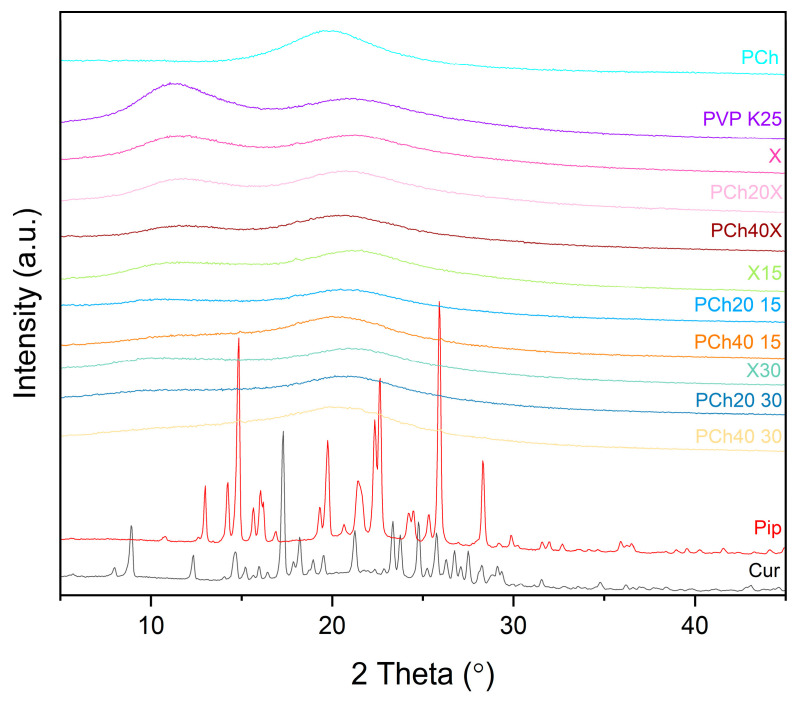
XRPD analysis of curcumin, piperine, and extruded amorphous solid dispersions, as well as modified excipients and phospholipids.

X-ray powder diffraction (XRPD) analysis was performed to confirm the amorphous nature of the dispersions obtained. XRPD patterns of raw compounds exhibited their crystalline nature with characteristic peaks at 8.92°, 12.35°, 14.65°, 17.29°, 18.20°, 21.25°, 23.34°, 23.76°, and 24.77° 2 theta, for curcumin and at 12.98°, 14.26°, 14.87°, 15.63°, 16.05°, 19.87°, 21.46°, 22.34°, 22.64°, 25.92°, and 28.31° 2 theta for piperine. Phospholipids showed an amorphous pattern. XRPD analysis of the fabricated systems showed a halo effect. The absence of peaks in the diffractograms suggests that the active compounds lost their original crystalline character and transformed into an amorphous state due to their dispersion in the polymer–phospholipid matrix (Figure 3).

**Figure 4:**
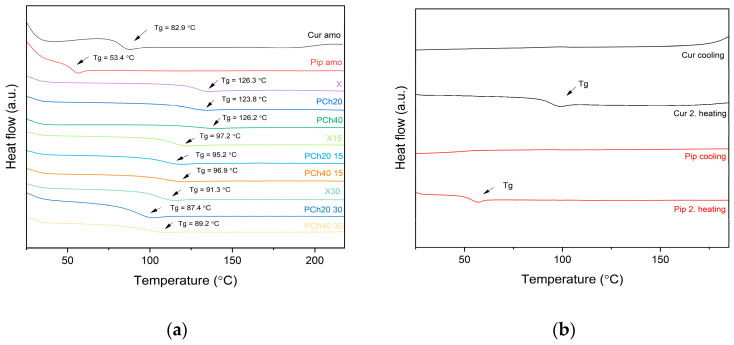
DSC thermograms of amorphous raw compounds, obtained dispersions, and modified excipients (**a**). The glass-forming ability of curcumin and piperine. Cooling curves represent a 2 °C/min cooling rate (**b**). Arrows point at Tg values.

Continuing the solid-state characterization, we performed DSC analysis to determine the thermal properties and estimate the Tg values. The Tg is an important parameter in assessing the miscibility and the stability of amorphous systems. DSC thermograms (Figure 4a) indicated the occurrence of a single Tg of the systems, indicating their homogeneity and good miscibility [41]. Moreover, the lack of endothermic peaks corresponding to the melting temperatures of compounds (curcumin Tm = 184.0 °C; piperine Tm = 133.1 °C [42]) supports the XRPD results and confirms the amorphous state of the dispersions. It can be seen that the Tg values of the systems were intermediate values between the Tg values of the active compounds and excipients. The polymer, as the component with the highest Tg value, acted as an anti-plasticizer to the other components of the dispersion. In contrast, curcumin, piperine, xylitol, and phospholipids decreased the Tg of the polymer, so they showed plasticizing properties. It should be noted that the polymer, exhibiting an anti-plasticizer effect, will ensure the physical stability of the systems, reducing the mobility of molecules and thus inhibiting recrystallization [43]. Higher Tg values are associated with better physical stability during storage and processing [12]. Moreover, we assessed the glass-forming ability of curcumin and piperine to estimate the crystallization tendency of both compounds. This study was performed using a heating–cooling–heating program by applying different cooling rates (10 °C/min, 5 °C/min, and 2 °C/min) as suggested by other researchers [44]. The results indicated the good glass-forming ability of curcumin and piperine since thermograms showed no crystallization (exothermic peak) during cooling and following heating (Figure 4b) [45].

**Figure 5:**
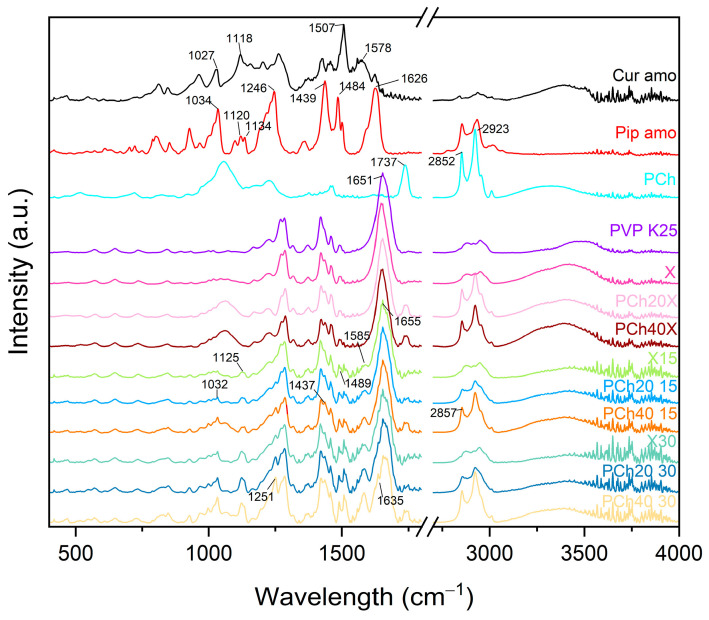
FTIR-ATR spectra of amorphous raw compounds, excipients, carriers, and amorphous solid dispersions.

To provide more information about the interactions formed in developed systems, the Fourier-Transform Infrared Spectroscopy (FT-IR) analysis was performed (Figure 5). Comparing the spectra of carriers with unmodified PVP K25 and phospholipids, one can note some changes that indicate interactions in the modified carrier. The 2852 cm^−1^ band of phospholipids shifted to 2855 cm^−1^ in the obtained carrier, and the 1737 cm^−1^ band of phospholipids shifted to 1742 cm^−1^ in the carrier. 

The spectra of systems were compared with amorphous forms of curcumin and piperine. The changes in the crystalline and amorphous form spectra of plant compounds were previously reported [42]. Owing to the presence of OH and C=O groups in the curcumin molecule, it can be both a donor and an acceptor of protons in a hydrogen bond. In contrast, the piperine molecule’s C=O group makes it a potential hydrogen bond acceptor. The spectra of the systems were dominated by bands from PVP K25, which was most abundant in the dispersion. Systems with phospholipids show a shift in the band from 2852 cm^−1^ for phospholipids to 2857 cm^−1^. In contrast, the band at 1737 cm^−1^ from phospholipids disappeared in systems with 20% phospholipids, with 40% phospholipids having a weaker intensity. The spectra of amorphous dispersions with and without phospholipids were similar, except for the presence of characteristic bands for phospholipids, 2855 cm^−1^ and 1742 cm^−1^ (band values from phospholipids in the modified carrier). The band at 1651 cm^−1^ from PVP K25 moved in the systems to 1655 cm^−1^. In contrast, the band at 1626 cm^−1^ for piperine can be seen as a small peak at 1635 cm^−1^ on the slope of the peak at 1655 cm^−1^. The band at 1585 cm^−1^ of the systems corresponds to the peak at 1578 cm^−1^ from curcumin, which shifted. The band at 1489 cm^−1^ of the system may be a shifted band of piperine at 1484 cm^−1^ or curcumin at 1507 cm^−1^. The band at 1437 cm^−1^ may be a shifted band of piperine at 1439 cm^−1^, and the band at 1251 cm^−1^ is a shifted band of piperine from 1246 cm^−1^. The band with an absorption maximum of 1125 cm^−1^ is probably a merged band from piperine at 1120 cm^−1^ and 1134 cm^−1^ and curcumin at 1118 cm^−1^. The peak at 1032 cm^−1^ in the scatterers could be a piperine peak at 1034 cm^−1^ or a curcumin peak at 1027 cm^−1^. The observed peak shifts confirm the occurrence of interactions between the components of the systems.

**Figure 6:**
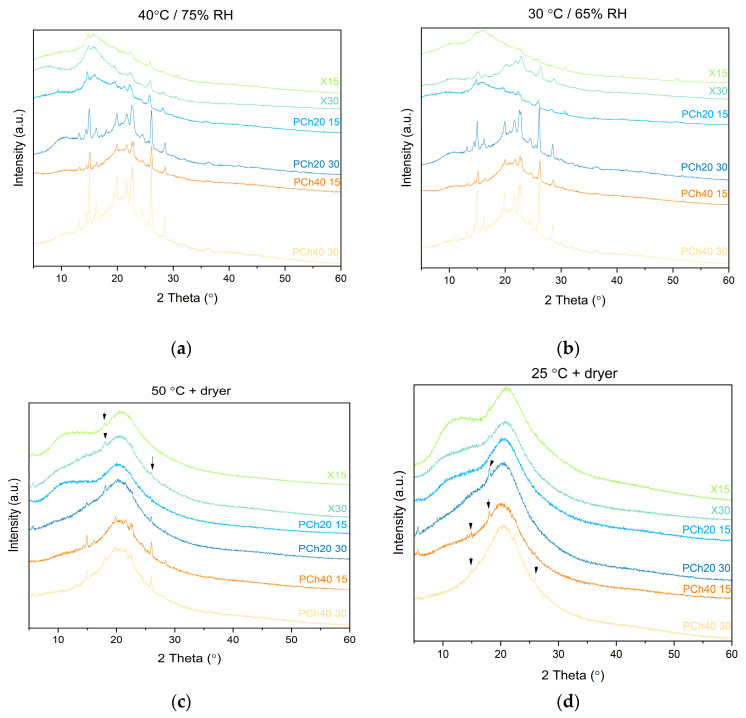
The results of stability studies. XRPD diffractograms of dispersions stored at different conditions: 40 °C/75% RH (**a**), 30 °C/65% RH (**b**), 50 °C/dry (**c**), and 25 °C/dry (**d**).

As part of the examination of the physicochemical properties relevant to the applicability of the obtained systems, we conducted physical stability tests to see how the different storage conditions would affect the stability of the amorphous form. The test was carried out under increased humidity (75% and 65%) and elevated temperatures (30 °C, 40 °C, and 50 °C). After 9 months, an XRPD test was performed to assess whether the amorphous form was preserved. The results of the stability tests are shown in Figure 6. Diffractograms showing the samples stored at 40 °C and 75% RH and at 30 °C and 65% RH show a clear mixing of the amorphous and crystalline phases, suggesting the occurrence of crystallization in all samples. On the other hand, under moisture-limited conditions at 50 °C, crystallization occurred in samples PCh20 30, PCh40 15, and PCh40 30. In contrast, small reflexes at 18.20° or 25.92° in X30 could be seen in samples X15 and X30, which may suggest the beginnings of crystallization. Sample PCh20 15 showed a typical amorphous diffractogram. Diffractograms of systems stored at 25 °C with limited moisture showed an amorphous pattern, but in the case of PCh20 30, PCh40 15, and PCh40 30, the small peaks may be due to an initiated crystallization process. 

The results of this study show that storage conditions are crucial to the shelf life and the potential use of the systems in the final drug formulation and its shelf life. Most relevant is the limitation of moisture access. It is indicated that humidity has a key effect on the shelf-life of amorphous products. Water, acting as a plasticizer, lowers the value of the glass transition in an uncontrolled way, which creates conditions for greater molecular mobility in the system and promotes crystallization [46,47]. Given that phospholipids have an amphiphilic character, i.e., are partially hydrophobic, we hypothesized that these systems would have greater moisture resistance than systems with only PVP K25 as the carrier. From visual assessment, it seemed that these systems did not absorb as much moisture as those with the polymer alone, which liquefied, looking like a “molten” material. The dispersions containing the phospholipid additive looked like solid chunks. However, as the diffractograms show, crystallization occurred under elevated humidity conditions in all samples. Amorphous dispersions based on hydrophilic polymers are much more sensitive to moisture. Lehmkemper et al. reported that under dry storage conditions, PVP K25 had the highest stabilization capacity of the amorphous forms of the investigated active substances, while in the presence of moisture, the stabilization ability drastically decreased, which was influenced by the hydrophilicity of the polymers [13]. In this case, the best stability under high humidity conditions was shown by dispersions based on the least hydrophilic polymer among those tested, HPMCAS. Moreover, the technique itself for obtaining amorphous dispersions is expected to affect the stability of the systems due to the effect on the moisture content of the obtained systems. It is most advantageous to use methods based on elevated temperature, as the process eliminates moisture due to evaporation, while techniques involving the preparation of solution, such as freeze-drying or spray-drying, favor a higher moisture content in the obtained dispersion, which affects physical stability [48].

The goal of our research was to obtain amorphous polymer–phospholipid dispersions to improve solubility and permeability. Therefore, in the next step, we expanded our research to determine the biopharmaceutical properties. As amphiphilic structures, phospholipids can self-organize in aqueous solution, where they form micelle-like structures. When administered with a lipophilic drug, phospholipids form a micelle-like structure where the drug molecule will be encapsulated in a phospholipid matrix [49,50]. These self-organized complexes can advantageously affect solubility and permeability. We first carried out solubility studies of the produced systems. The results of this study are shown in Table 2.

Solubility testing was carried out at a pH of 1.2 to simulate the stomach environment and a pH of 6.8 to represent the physiological conditions in the intestine. The obtained solubility of the systems was compared with that of crystalline compounds to determine the extent of the improved parameter. Based on the results of the solubility test, curcumin and piperine could be classified as practically insoluble compounds, with curcumin dissolving more poorly than piperine. As can be seen in Table 1, the dispersions produced led to a remarkable improvement in solubility. The best system improved the solubility of curcumin 17009-fold under acidic conditions and 25593-fold in phosphate buffer, while for piperine, it was 209- and 447-fold, respectively. Drug load affected solubility. Systems with lower amounts of active compounds generated higher concentrations. This may be related to the higher amount of supersaturation state stabilizers (polymer and phospholipid) per compound of molecule in the case of a lower drug load, which promoted a more effective stabilization of the amorphous form in solution and the generation of a higher supersaturation state. Moreover, the presence of phospholipids in dispersion and, therefore, in solution, affected curcumin and piperine concentrations. The best improvement in solubility was noted for systems containing a 20% phospholipid addition. This observation can be explained by the solubilizing effect of the phospholipid. The amphiphilic nature of the phospholipid molecule caused the self-assembling and entrapment of polyphenol molecules in micelles to occur. Interestingly, a higher number of phospholipids did not promote a greater improvement in solubility, suggesting there is optimal phospholipid content in the dispersion, which, through some synergistic action with the polymer, maximizes the supersaturation state. It can be speculated that the phospholipids act as boosters of the supersaturated state, allowing for an even greater improvement in solubility than if only the polymer were used as a stabilizer of the amorphous form after the system is dissolved. This may be related to the direct interaction of the phospholipid with the poorly soluble compound since the compound molecule and the phospholipid tail exhibit a similar hydrophobic character. The contribution of the polymer, which, through various mechanisms such as slowing down and hindering crystallization, cannot be overlooked [51,52]. 

**Figure 7:**
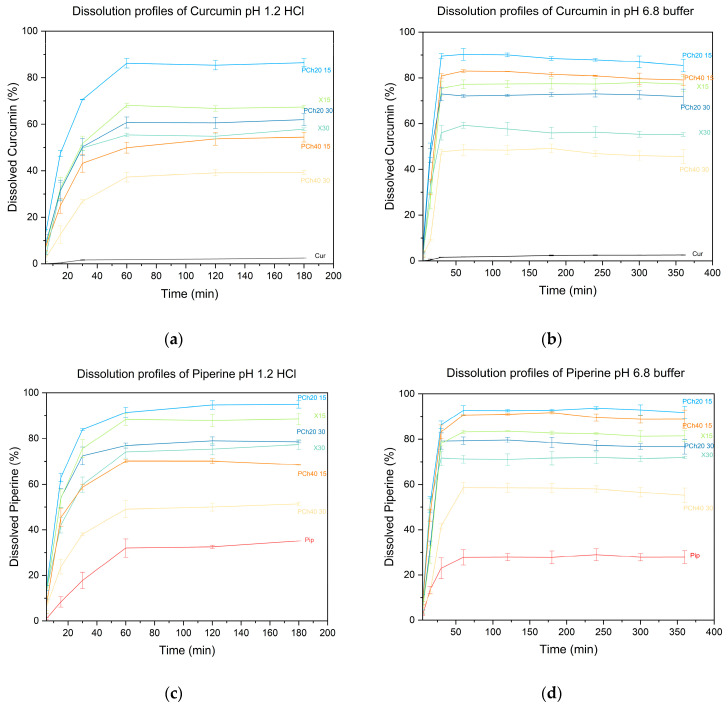
Dissolution rate profiles for amorphous systems of curcumin in pH 1.2 HCl (**a**) or pH 6.8 buffer (**b**) and piperine in pH 1.2 HCl (**c**) or pH 6.8 buffer (**d**).

Proceeding with our experiments, we performed dissolution studies to determine dissolution profiles and estimate whether we would observe the maintenance of the supersaturated state or observe decreases in the number of dissolved compounds over time. In other words, we wanted to see if we could observe a spring and parachute effect and how effective a parachute formed by a phospholipid and a polymer is. The spring refers to the rapid dissolution of the system and the release of the active ingredient from the dispersion and the generation of a supersaturated state, while the parachute refers to the maintenance of supersaturation, which creates the possibility for increased absorption of the active ingredient [53]. The release profiles of the obtained amorphous dispersions are shown in Figure 7.

The release profiles showed an increase in the amount of active substance in the medium, which reaches a maximum in the range of 30–60 min. The observed phenomenon is due to the dissolution of the gelatin capsule in which we placed the powder before testing, as well as the dissolution of the amorphous dispersions. The systems were able to reach a supersaturated state, as a higher amount of dissolved active compounds than crystalline compounds was observed. In addition, one can see the maintenance of the plateau state for the test period, with no clear decreases in the solubility of the compounds, indicating an effective parachute effect of the obtained systems. The polymers and phospholipids present in the dispersions effectively inhibited the phenomenon of crystallization of active substances from the solution. It is known that the metastable amorphous form readily transforms into a stable crystalline form during dissolution, which is the main factor sabotaging the beneficial effect of amorphization on bioaccessibility because when it occurs, leverage is given by the amorphous form in terms of increased solubility; thus, enhanced passive absorption is lost [54]. Moreover, this study confirmed observations from solubility studies. In general, the individual systems achieved higher solubility in a phosphate buffer with a pH of 6.8 than in acidic conditions with a pH of 1.2.

The solubilizing and stabilizing effect on the supersaturation state of phospholipids was demonstrated by Fong et al. in a paper on the amorphous dispersion of celecoxib in a matrix of phospholipids. The obtained values of apparent solubilities were improved compared to crystalline and amorphous forms of the drug; this showed that phospholipids enhanced the solubility improvement effect provided by the amorphous form. Moreover, permeation tests showed that throughout the test period, the amount of active ingredient that permeated increased at each time point, demonstrating the maintenance of the drug’s supersaturated state, which was responsible for increased diffusion through the membrane [55].

Two components are present in the obtained systems that will be responsible for inhibiting crystallization and maintaining the supersaturation state: the polymer and the phospholipid. The possible beneficial effect of phospholipids on supersaturation has already been mentioned. The role of the polymer should be emphasized.

The main carrier of the obtained amorphous dispersions is PVP, which is expected to demonstrate the stabilizing role of the amorphous form, affecting the maintenance of the supersaturation state and also providing an acceptable shelf-life. The addition of polymers in the solution can prevent the crystallization of active compounds from supersaturated solution or reduce the crystallization rate, extending the effective supersaturation window and providing the opportunity for enhancing permeability [56]. In their study, Knopp et al. prepared an amorphous dispersion of celecoxib with PVP. They observed a significant increase in the dissolution rate and the obtaining of a supersaturated state compared to the drug in crystalline form. Moreover, the supersaturated state was maintained during the 24 h study. In the rat oral administration model, the amorphous dispersion showed superiority over the crystalline drug, resulting in a higher plasma concentration of celecoxib, thus presenting an advantageous effect of amorphous dispersion [57]. The use of the polymer delayed crystallization, prolonging the supersaturated state and resulting in improved bioavailability. In another paper, Dahan et al. reported on obtaining an amorphous dispersion of nifedipine using spray drying. The resulting formulation improved the solubility of the active compound by 20-fold and improved the bioavailability of nifedipine in an in vivo study in rats [58]. At this point, it is worth noting that we used PVP K25 as the polymer. Nogami et al. indicated PVP as a superior polymer in curcumin systems, providing a great improvement in solubility [59].

Continuing our research, we moved on to investigate whether improving solubility would have a beneficial effect on the permeability of curcumin and piperine. We performed the test in an in vitro model simulating passive absorption. Table 3 shows the concentrations of active compounds obtained in the acceptor part of the model. In this study, we determined the effect on absorption in the gastrointestinal tract and blood-brain barrier models.

The results of this study indicate a better permeability of the tested compounds through artificial biological membranes than crystalline forms of compounds, which is associated with a significant improvement in solubility. Due to the state of supersaturation, the fraction of free molecules that can be absorbed increased. The bioaccessibility improved. Moreover, the highest concentration values in the acceptor were reached by the systems for which the greatest solubility enhancement was obtained. The improvement in permeability was remarkable as for the best system, it was 1193-fold and 2644-fold in the gastrointestinal tract and blood-brain barrier models for curcumin, while for piperine, it was 172-fold and 114-fold, respectively.

The disadvantage of in vitro permeation tests should be noted, where the permeability of a molecularly dissolved drug is verified, and the formation of supramolecular assemblies of phospholipids with the drug negatively affects the permeability. In the work of Fong et al., higher ratios of phospholipid to drug (50:1 or more) in the in vitro permeation test had the effect of lowering permeation despite a significant improvement in the solubility of the resulting amorphous dispersions compared to the crystalline drug. The authors explained this observation by the fact that drug molecules were entrapped in supramolecular assemblies that were not able to pass the membrane pores due to their size, as well as the flow release of the drug from those assemblies. As a result, these factors led to limitations in permeation [55]. In our study, the permeation results were in line with the solubility results; hence, we do not expect that the above phenomenon had a significant effect on the observations we obtained. This may be because the amount of phospholipid per molecule of the active compound did not exceed the limit, after which we would observe a decrease in permeability. The investigation by Fong et al. showed that the drug/phospholipid ratio is crucial.

In another study, Jacobsen et al. obtained phospholipid dispersions of celecoxib that showed improved in vitro permeability, which the authors attributed to the amorphous state of celecoxib that induced supersaturation. The observed permeation enhancement was linked to the encapsulation of poorly soluble celecoxib into micelles and liposomes, stabilizing the amorphous state and prolonging the supersaturation [50]. The research indicates that phospholipids can stabilize the amorphous state and associated supersaturation, having a beneficial impact on the permeation and thus can improve bioavailability. Furthermore, in in vivo studies, the formation of micelles or other phospholipid structures entrapping active compound molecules promotes absorption through a chylomicron-forming mechanism. Shi et al. obtained an amorphous dissolution of berberine with hydrogenated phosphatidylcholine, which led to improved concentrations of the active compound in rat plasma. Interestingly, dissolution studies showed comparable profiles and cumulative dissolution percentages of crystalline berberine and those complexed with phosphatidylcholine, suggesting that other factors controlled the enhancement in bioavailability rather than solubility [60]. This paper provides evidence that phospholipids can enhance the permeability of active components in vivo.

The phospholipid–active substance complex is absorbed by a similar mechanism to triglycerides or essential phospholipids. Phospholipids with cholesterol and bile salts can form mixed micelles, increasing the absorption of lipophilic substances. The generated micelles are absorbed into enterocytes by passive diffusion, resulting in the formation of a chylomicron containing the active substance. The chylomicrons enter the lymphatic vessels and are then transported to the systemic circulation, avoiding the first pass-effect [61,62]. Interestingly, phosphatidylcholine enhances lymphatic lipid transport. Thus, it can be speculated that this further boosts the absorption of the formed lipid particles containing the active ingredient into the lymphatic system [15].

The addition of a phospholipid into the formulation is intended to boost solubility and permeability. The increase in solubility occurs through a solubilization mechanism. Self-assembling drug–phospholipid complexes are formed in solution. We expect that this mechanism is more likely responsible for increasing but also maintaining the supersaturation state than the mechanism provided by the polymer. The hydrophobic part, which can be distinguished in the structure of the phospholipid, is more probable to interact with the hydrophobic molecule of active substances. However, the polymer affects the maintenance of the supersaturated state by hindering crystal nucleation and slowing crystallization. In turn, enhancement in permeability is conditioned by high biocompatibility and similarity in structure to the cell membrane of phospholipid micelles in which the compound molecule is trapped. In addition, enabling the transport of the active ingredient through the chylomicron into the lymphatic system enhances the bioavailability of active substances [63].

It is important to recognize the limitations of this work. The phospholipid employed in the research was predominantly phosphatidylcholine, with a relatively low purity (21%); therefore, the observed effect of improving biopharmaceutical parameters can be attributed not only to phosphatidylcholine but also to other substances present in the excipient.

In this work, we developed a solubility-enabling formulation, suggesting a way to obtain amorphous polymer–phospholipid dispersions by hot-melt extrusion. The main factor limiting the therapeutic potential of curcumin and piperine is poor bioavailability. These plant-origin compounds can be classified as Class II representatives in the biopharmaceutical classification system; hence, the main obstacle to overcome was poor solubility. The compounds show good permeability, with a Log P of 3.28 and 2.78 for curcumin [64] and piperine [65], respectively. The obtained dispersions, due to the amorphous state of the compounds, generated a supersaturated state which boosted the permeability of the compounds in the passive diffusion model. Based on the literature, it can be speculated that the phospholipid will promote the absorption of the compounds in vivo.

## 4. Conclusions

In this study, we focused on obtaining amorphous dispersions of curcumin–piperine combinations using a polymer–phospholipid matrix. Amorphous polymer–phospholipid dispersions used as enabling formulations are a relatively new concept in delivery systems, so their potential is still not fully explored, so we were motivated to undertake our research. The novelty of the work is also a demonstration that these kinds of formulations can be produced by hot-melt extrusion. The phospholipid showed a plasticizing effect on PVP K25; however, it was too poor. Therefore, it was necessary to add xylitol with a considerably higher plasticizing potential on PVP K25. This approach provided the desired thermoplastic properties of the polymer–phospholipid blend and allowed for a smooth hot-melt extrusion process, resulting in an amorphous dispersion of curcumin and piperine. 

We showed that the developed systems lead to improved solubility and permeability. The observed enhancement in the performance of the dispersions as compared to a crystalline form of curcumin and piperine is connected to conversion to an amorphous state, which induced the supersaturation. It appears the amorphous systems inhibit crystallization and thus prolong the supersaturation window. To the best of our knowledge, the effects, such as the amorphous form of drugs and the formation of supramolecular structures that encapsulate the molecules of active compounds, may be responsible for enhanced solubility. In addition, the increase in solubility is associated with the presence of a phospholipid acting as a solubilizer and stabilizer of the amorphous state. Moreover, we found an increase in permeability in an in vitro model, and the results were linked to solubility results. Increasing solubility generated an improvement in permeability by creating a concentration gradient that stimulates passive transport.

## Figures and Tables

**Table 1:** The composition of formulations.

Name	Percentage of Ingredient			
	Actives	Polymer	Xylitol	Phospholipid
X30	30	63.83	6.17	0
PCh20 30	30	48.27	1.73	20
PCh40 30	30	29.14	0.86	40
X15	15	77.51	7.49	0
PCh20 15	15	62.75	2.25	20
PCh40 15	15	43.71	1.29	40

**Table 2:** The results of solubility studies of amorphous dispersions are presented as achieved concentrations and folds of improvement of curcumin and piperine.

			Compound			
			Curcumin		Piperine	
			Conc. [mg/mL]	Improv. [-fold]	Conc. [mg/mL]	Improv. [-fold]
**System**	pH 1.2 HCl	Raw	0.00011 ± 0.00001 ^e^	N/A	0.009 ± 0.0006 ^e^	N/A
		X30	1.112 ± 0.091 ^c^	10109	1.082 ± 0.084 ^c^	120
		PCh20 30	1.243 ± 0.102 ^c^	11300	1.193 ± 0.107 ^c^	133
		PCh40 30	0.468 ± 0.076 ^d^	4255	0.456 ± 0.061 ^d^	51
		X15	1.637 ± 0.113 ^b^	14881	1.592 ± 0.119 ^b^	177
		PCh20 15	1.871 ± 0.126 ^a^	17009	1.881 ± 0.123 ^a^	209
		PCh40 15	0.536 ± 0.082 ^d^	4873	0.524 ± 0.077 ^d^	58
	pH 6.8 Phosphate buffer	Raw	0.00014 ± 0.00002 ^e^	N/A	0.008 ± 0.0004 ^f^	N/A
		X30	1.155 ± 0.109 ^c^	8250	1.129 ± 0.098 ^d^	141
		PCh20 30	2.325 ± 0.114 ^b^	16607	2.347 ± 0.121 ^c^	293
		PCh40 30	0.618 ± 0.082 ^d^	4414	0.594 ± 0.074 ^e^	74
		X15	2.482 ± 0.137 ^b^	17729	2.471 ± 0.108 ^b,c^	309
		PCh20 15	3.583 ± 0.116 ^a^	25593	3.576 ± 0.123 ^a^	447
		PCh40 15	2.528 ± 0.099 ^b^	18057	2.533 ± 0.086 ^b^	317

The statistically significant values are presented as “^a–f^”, with “^a^” being the highest value and “^f^” being the lowest (*p* < 0.05). N/A means not applicable.

**Table 3:** The results of the in vitro permeability assay for raw curcumin and piperine and those from amorphous dispersions.

			Compound			
			Curcumin		Piperine	
			Conc. [mg/mL]	Improv. [-fold]	Conc. [mg/mL]	Improv. [-fold]
Model	GIT	Raw	0.00061 ± 0.000038 ^g^	N/A	0.0044 ± 0.0008 ^g^	N/A
		X30	0.310 ± 0.012 ^e^	508	0.367 ± 0.016 ^e^	83
		PCh20 30	0.421 ± 0.013 ^d^	690	0.455 ± 0.025 ^d^	103
		PCh40 30	0.134 ± 0.012 ^f^	220	0.282 ± 0.018 ^f^	64
		X15	0.547 ± 0.023 ^c^	897	0.594 ± 0.022 ^c^	135
		PCh20 15	0.728 ± 0.051 ^a^	1193	0.757 ± 0.038 ^a^	172
		PCh40 15	0.651 ± 0.028 ^b^	1067	0.673 ± 0.021 ^b^	153
	BBB	Raw	0.00016 ± 0.000057 ^f^	N/A	0.0051 ± 0.0003 ^f^	N/A
		X30	0.178 ± 0.025 ^d^	1113	0.358 ± 0.034 ^d^	70
		PCh20 30	0.269 ± 0.023 ^c^	1681	0.409 ± 0.014 ^c^	80
		PCh40 30	0.098 ± 0.018 ^e^	613	0.263 ± 0.021 ^e^	52
		X15	0.274 ± 0.011 ^c^	1713	0.427 ± 0.013 ^c^	84
		PCh20 15	0.423 ± 0.028 ^a^	2644	0.583 ± 0.029 ^a^	114
		PCh40 15	0.346 ± 0.022 ^b^	2163	0.471 ± 0.024 ^b^	92

The statistically significant values are presented as “^a–g^”, with “^a^” being the highest value and “^g^” being the lowest value (*p* < 0.05). N/A means not applicable.

## Data Availability

Data are contained within the article.

## Supplementary Materials

The following supporting information can be downloaded at: https://www.mdpi.com/article/10.3390/pharmaceutics16080999/s1, Table S1: HPLC method validation parameters. Figure S1: Chromatograms of standards and systems, curcumin standard (a) and piperine standard (b).
