# Supplementary material for: Amorphous Polymer–Phospholipid Solid Dispersions for the Co-Delivery of Curcumin and Piperine Prepared via Hot-Melt Extrusion
Source: Pharmaceutics. 2024 Jul 28;16(8):999. doi: 10.3390/pharmaceutics16080999 (PMC11359794; doi:10.3390/pharmaceutics16080999)
Supplement: Supplementary file 1 [file pharmaceutics-16-00999-s001.zip › pharmaceutics-3107977-supplementary.pdf]

pharmaceutics

Supplementary Materials: Amorphous Polymer–Phospholipid Solid Dispersions for the Co-Delivery of Curcumin and Piperine Prepared via Hot-Melt Extrusion

Kamil Wdowiak, Andrzej Miklaszewski and Judyta Cielecka-Piontek

Chromatographic conditions:
- Stationary phase – Dr. Maisch ReproSil-Pur Basic-C18 100 Å column, 5 µm particle size, 100 × 4.60 mm
- Mobile phase – methanol/0.1% acetic acid (85:15 *v*/*v*)
- Column temperature – 30 °C
- Flow rate – 0.3 mL/min

Table S1. HPLC method validation parameters.

Curcumin	
Parameter	Curcumin dissolved in methanol<br>Injection volume 10 µL
Linearity range (mg/mL)	0.000024–0.048
Correlation coefficient (r)	0.9997
$a \pm S_a$	335533780 ± 6181899
$b \pm S_b$	insignificant (α = 0.05)
LOD (mg/mL)	0.0013
LOQ (mg/mL)	0.0041
Retention Time	4.912
**Piperine**	
Parameter	Piperine dissolved in methanol<br>Injection volume 10 µl
Linearity range (mg/mL)	0.0000048 – 0.048
Correlation coefficient (r)	0.9999
$a \pm S_a$	303555921 ± 2091025
$b \pm S_b$	insignificant (α=0.05)
LOD (mg/mL)	0.0005
LOQ (mg/mL)	0.0015
Retention Time (min)	5.851

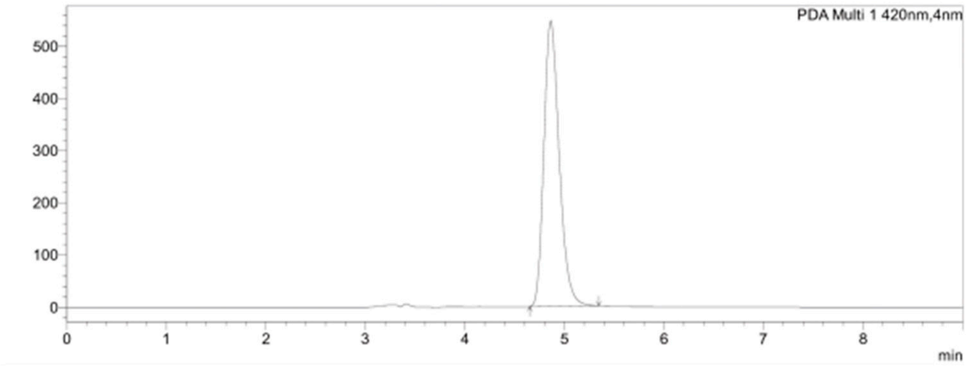

(a)

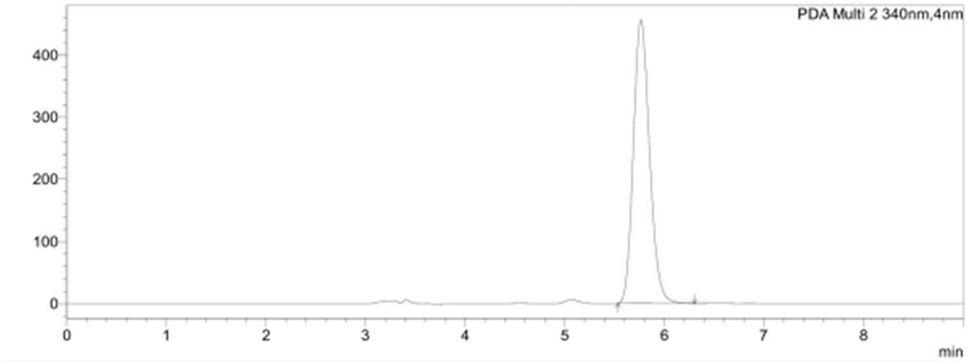

(b)

Figure S1. Chromatograms of standards, curcumin standard (a), piperine standard (b).
